# Vibration-Induced-Flow Mechanism and Its Application in Water Surface Robot

**DOI:** 10.34133/research.0449

**Published:** 2024-08-09

**Authors:** Dehong Wang, Shijing Zhang, Jing Li, Haoxuan He, Weishan Chen, Junkao Liu, Jie Zhao, Jie Deng, Yingxiang Liu

**Affiliations:** State Key Laboratory of Robotics and System, Harbin Institute of Technology, Harbin 150001, China.

## Abstract

Vibration is a common strategy for aquatic organisms to achieve their life activities, especially at the air–water interface. For the locomotion of small creatures, the organs with plate features are often used on water surfaces, which inspires relevant studies about using thin plates for robot propulsions. However, the influence of the general deformations of thin plates on the generated flow fields has not been considered. Here, a comprehensive investigation is conducted about the flow fields that arose by vibrations of thin plates and the potential as locomotion strategies are explored. It is discovered that as thin plates are subjected to vibration excitations on the water surface, the produced flow fields are mainly determined by the vibration shapes, and the influence rules of plate deformations on the flow fields are identified. The main factors producing asymmetric flow fields are analyzed to realize the morphology control of the flow fields. Then, to determine effective locomotion strategies on the water surface, the flow fields stimulated by integrated vibration exciters are explored, and 2 water surface robots are developed consequentially, which exhibit superior motion performance. This work reveals the basic rules of the vibration-induced-flow mechanism by thin plates and establishes new locomotion strategies for aquatic robots.

## Introduction

Vibration is an important physical phenomenon as well as a biological process in natural environments, and many organisms interact with fluids, including air and water, through vibrations to control the movement of fluid clusters and then realize their life activities such as object-harvest [1] and self-propulsion [2,3]. For miniature creatures that can propel on the air–water interface, a typical locomotion method based on vibration is adopted via the traveling waves along their organs or bodies [4,5]. It inspires relevant studies focusing on the realization of traveling waves based on strip-type thin plates with only bending deformations [6], while the more general locomotion cases of thin plates, indicated by the wings of insects such as dragonflies or honeybees [7], get less attention. Actually, when a typical thin plate vibrates on the water surface, different deformations will inevitably arise in the form of vibration shapes and the specific deformations of the plates can produce stable flow fields on the water surface. However, the generation mechanism of these induced flow fields is not clear enough, and there are few studies adopting this mechanism for aquatic locomotion.

The locomotion strategy on the water surface is one of the most popular research topics, and many impressive miniature robots inspired by different strategies have been developed recently. A common locomotion strategy is based on surface tension by imitating striders [8–10], and the robots can achieve walking [11] or jumping [12–14] on water surfaces owing to their rod-like legs. The leg can also be designed with special materials or active actuation ability [15] to increase the motion performance. However, they generally cannot generate strong propulsion forces. Another typical locomotion strategy is based on the Marangoni effect. It creates a surface tension gradient by surfactant [16–20] or heating [21,22] for prolusion, but endurance and contamination issues are usually unavoidable. The electrowetting-on-dielectric method can also be used for water surface locomotion [23], which arranges the electrodes at the end to realize the movement by voltage excitation [24,25], while it is generally a challenge to be detached from the constraints of wires for the requirement of external power supplies. Regarding the locomotion strategy based on thin plates, the propulsions are mostly realized by the travelling waves and the robots usually adopt strip-plate bodies fabricated by special material, which can generate expected deformations excited by light power [26–28] or magnetic field [29,30]. However, their motion speeds are relatively slow, and the stimulation devices are difficult to integrate. The above locomotion strategies realize locomotion on water surfaces with features of small size, light weight, and fast speed, but the existing problems limit their practical applications. Therefore, a new locomotion strategy is required for the miniature water surface robots to realize the applications in real environments.

**Fig. 1.**
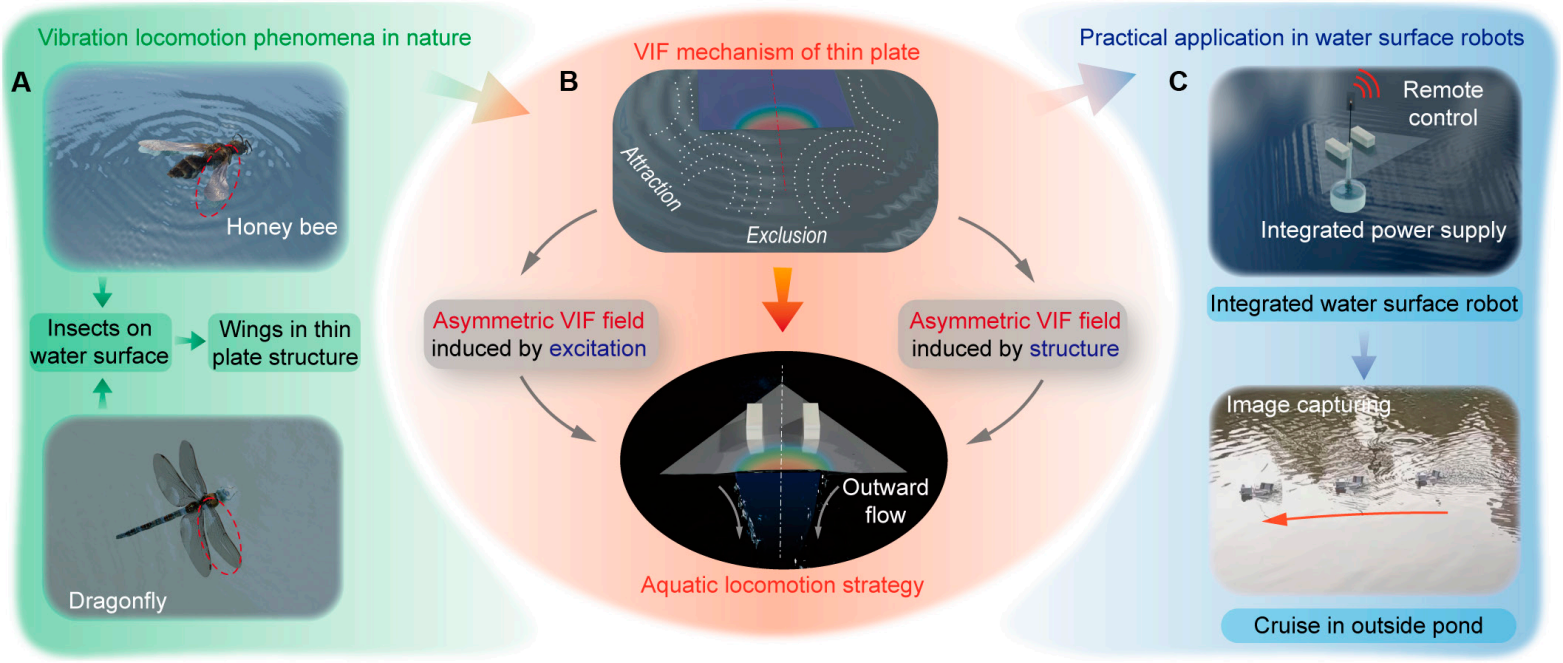
The VIF mechanism from natural phenomena to application. (A) Vibration locomotion phenomena in natural environments with organs of thin-plate structures. (B) Exploration of the VIF mechanisms and establishment of aquatic locomotion strategies. (C) Application of the VIF mechanism of water surface robots.

In this work, we conduct comprehensive research about the vibration-induced-flow (VIF) phenomena of thin plates on water surfaces and innovatively reveal the basic rules of the VIF mechanism when subjected to vibration excitations with general deformations. This mechanism is utilized to develop miniature water surface robots, which verifies its promising prospection as the aquatic locomotion strategy, as summarized in Fig. 1. The main contributions of this work are as follows: (a) A VIF mechanism between the thin plates and fluid motions on the water surface is revealed, and the influences of the general plate deformations on the flow fields are investigated. (b) The factors determining the asymmetric VIF fields are analyzed, and the generation and control methods of the VIF fields are proposed. (c) New locomotion strategies for water surface robots with high vibration frequencies are established based on the VIF mechanism, which provides foundations for the fast and agile motion of robots. (d) Two miniature water surface robots integrating power supply, control unit, wireless communication, and sensor module are developed. (e) The experiments in environment detecting and information monitoring are conducted, which demonstrates the application potential of the VIF mechanism.

## Results

### VIF mechanism based on thin plates

As objects vibrating on the water surface, the vibration can inject energy into the fluid in the vicinity continuously and induce the motion of fluid clusters, thus generating a specific flow field. This VIF phenomenon varies with the object structures and excitation conditions, especially for the objects that can be obviously deformed. The thickness of a thin plate is far less than its in-plane dimensions, so it can easily produce obvious out-plane deformation when subjected to normal vibration excitations on the water surface (see Note S1 and Fig. S1).

**Fig. 2.**
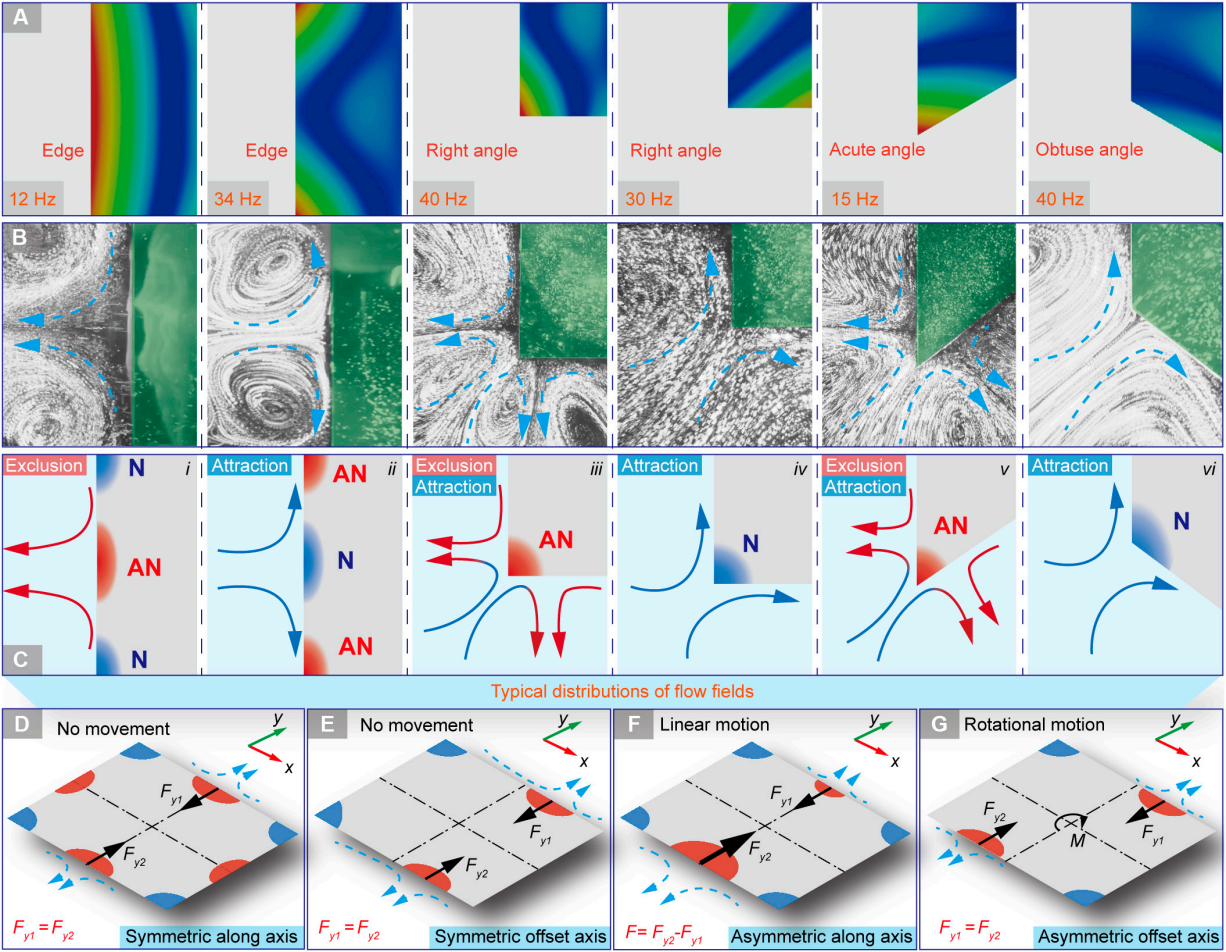
The influence of the edges and angles of a thin plate on flow fields. (A) Simulated deformations of typical regions on thin plates. (B) Corresponding flow fields of the typical regions on the thin plates in experiments. (C) Mechanism of the VIF principle. (D) Symmetric flow field along axis. (E) Symmetric flow field offset axis. (F) Asymmetric flow field along axis. (G) Asymmetric flow field offset axis.

To identify the influence on the VIF fields raised by localized deformations of different regions (edges and angles) on thin plates, we analyze the harmonic response characteristics of typical thin plates on the water surface, as shown in Fig. 2A. Then, we conduct the observation experiments of the flow fields induced by plate vibrations, as shown in Fig. 2B. The results show that the regions with different deformations exhibit divergent influence on the surrounding flow field, and it mainly manifests as 2 different effects: the attraction and the exclusion of fluid clusters (see Movie S1). For the edges of thin plates (see i and ii in Fig. 2C), the wave anti-node regions (red regions marked as AN) exhibit as exclusion of the flow field vertical to the edge as well as attraction of the flow field along the edge, whereas the wave node regions (blue regions marked as N) exhibit the opposite phenomena. The angles of thin plates always exhibit as attraction of the flow field in the direction of the angular bisector (see iii and iv in Fig. 2C). However, the wave anti-node regions show exclusion of the flows in the perpendicular directions of 2 side edges nearby, whereas the flows just converge along 2 side edges for the wave node regions. The degree of angle will also affect the generation of flow fields (see v and vi in Fig. 2C). Acute angles are more prone to deform as anti-nodes than the obtuse angles, and the lateral components of outward flows will be greater for smaller angles due to the perpendicular direction of the outward flows.

As the vibration shapes can be regarded as standing waves, the vibration states of different positions on the plate are simple harmonic oscillations with definite amplitudes and the same frequencies. Then, the abovementioned attraction and exclusion flow fields are separately induced by node and anti-node regions due to their different vibration amplitudes, which indicates the basic rules of the VIF mechanism. Furthermore, the flow fields can be controlled by adjusting the distribution of the nodes and anti-nodes, that is, the vibration shape of the thin plates.

The distributions of the plate deformations can be concluded as 4 typical patterns: symmetric along axis, symmetric offset axis, asymmetric along axis, and asymmetric offset axis, as shown in Fig. 2D to G. The symmetric deformation patterns will produce compensated flow fields, and the influence of the flows in opposite directions cancels each other, producing zero resultant force. On the contrary, the asymmetric deformation patterns can induce asymmetric flow fields and thus non-zero resultant forces to realize motions. When a linear motion is required in a certain direction, the flow field along the direction is supposed to be asymmetric (while in other directions symmetric) to generate a unidirectional resultant force (see *F* in Fig. 2F). As a rotational motion is required around a certain axis, the flow field should be asymmetric in both directions to form a resultant moment (see *M* in Fig. 2G). We are expected to utilize this VIF mechanism for locomotion on the water surface, so the generation methods of specific asymmetric flow fields are analyzed in the following.

### Generation of asymmetric VIF fields

In general, the morphologies of flow fields for thin plates are mainly determined by 2 factors: plate structures and excitation conditions. Then, the influences of these 2 factors are analyzed individually to establish the generation and control methods of the asymmetric VIF fields.

#### Flow field analysis of typical thin plates with asymmetric structures

**Fig. 3.**
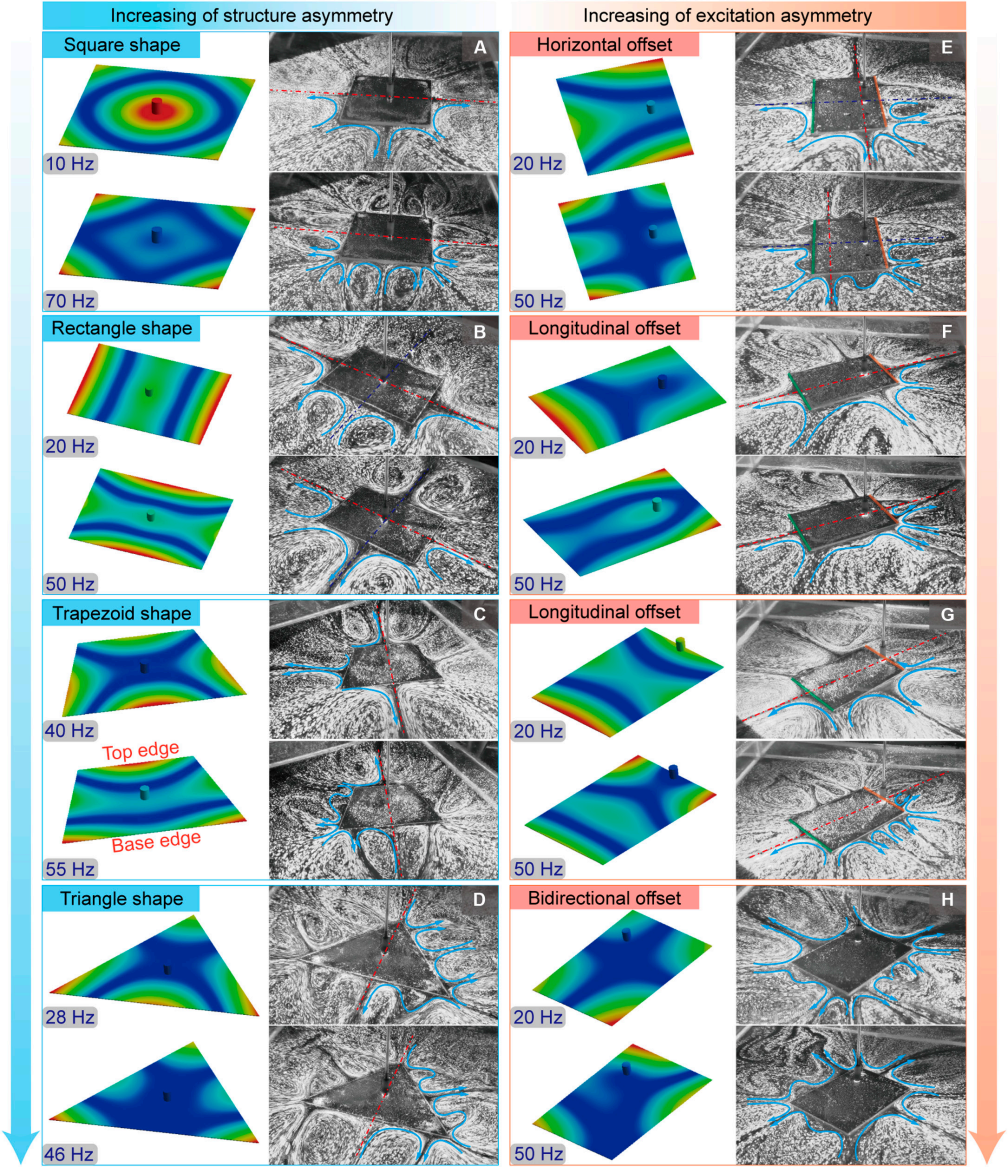
Flow fields generated by thin plates with asymmetric shapes and offset excitation positions. (A) Flow fields generated by square shape. (B) Flow fields generated by rectangular shape. (C) Flow fields generated by trapezoid shape. (D) Flow fields generated by triangular shape. (E) Flow fields generated by rectangular shape at horizontal offset excitation position. (F) Flow fields generated by rectangular shape at longitudinal offset excitation position. (G) Flow fields generated by rectangular shape at longitudinal offset excitation position (on edge). (H) Flow fields generated by rectangular shape at bidirectional offset excitation position.

To identify the influence of the asymmetric structures on the flow fields, a series of uniform plates with different shapes (square, rectangle, trapezoid, and triangle) are analyzed, in which the vibration excitations are set at geometric centers. The observed VIF fields of these plates at different frequencies in experiments, as well as the corresponding harmonic responses in simulations, are shown in Fig. 3A to D and Movie S2.

Although the flow fields are in well accordance with the basic rules of the VIF mechanism, the interaction of these node and anti-node regions is complicated, especially for vibration shapes at high frequencies. On the one hand, as these regions are in proximity of others, the produced flow fields are not distinct but connected with each other and function as part of the resultant flow field. On the other hand, the relative deformations at the node and anti-node regions are important for the interaction process. The regions with greater deformations exhibit more profound influence and even cancel the influence of small deformations. Here, we take the trapezoid plate (shown in Fig. 3C) as an example. At a frequency of 40 Hz, anti-nodes are shown in the middle of the top and base edges so that 2 obvious outward flows appear along the symmetry axis of the plate. Meanwhile, the anti-nodes are offset on side edges; thus, the outward flows are also offset to the base edge. At 55 Hz, the deformation in the middle of the base edge inverts to be a node region, resulting in flow fields in reverse directions. Furthermore, the anti-nodes in the middle of 2 side edges are compressed as vortexes for the small deformations. Despite the complicated deformations of plates at different frequencies, the resultant flow fields can always be recognized as a combination of node and anti-node region fields obeying the rules of the VIF mechanism.

As the length of one edge is reduced, the structure asymmetry of the thin plate is increased, and the plate shape will degenerate from a square or rectangle to a trapezoid and further to a triangle. Thus, the discrepancy of the flow field morphology increases along the symmetric axis. In detail, the morphologies of the flow fields for square plates are identical at all 4 edges, while the flow fields show discrepancy for rectangle plates at adjacent edges, in which the long edges and the short edges produce opposite flow fields, as shown in Fig. 3A and B. However, the flow fields remain symmetric along both axes. Regarding the trapezoidal plates, the inward or outward flows no longer appear symmetrically in pairs, and the flow fields can exhibit different strengths or even directions, as shown in Fig. 3C. For the triangular plate, the original top edge is reduced into an angle, and it shows distinct influences with edges, which always functions as attraction of flow fields in the bisector direction. Besides, as the number of edges is increased, the VIF fields will be complicated; this brings difficulties to the excitation and control of the fields (see regular triangular, regular hexagon, and ellipse in Note S2 and Fig. S2).

#### Flow field analysis on typical asymmetric excitation conditions

Another factor to generate the asymmetric flow field is the excitation condition. We take a rectangular plate as a sample to analyze the influence of the excitation position and frequency on the asymmetry of VIF fields. A harmonic excitation is exerted at positions offset the plate center (P_1_ located at one-quarter of horizontal axis *y*, P_2_ located at one-quarter of longitudinal axis *x*, P_3_ located at the end of longitudinal axis *x*, and P_4_ located at one-quarter of both *x* and *y* axes), as shown in Fig. S1. The VIF fields of the rectangular plate at different excitation positions are observed in experiments (see Movie S3), and the corresponding harmonic response simulations are carried out.

We adopt a rectangular plate with the same size and excitation frequencies as Fig. 3B for convenience of comparison. As the excitation is located at P_1_ (see Fig. 3E), the flow field is also offset compared with Fig. 3B, and the positions of the original pair of outward flows along longitudinal axis *x* change with the excitation positions and frequencies. Meanwhile, the deformation of the edge proximal to the excitation position (marked by orange line) is inhibited, while the opposite deformation (marked by green line) is enhanced, resulting in an outward flow on the distal side and an inward flow on the proximal side. Similar phenomena also appear when the excitation position is offset in positions of P_2_ and P_3_, as shown in Fig. 3F and G. The symmetric thin plate produces asymmetric flow fields under asymmetric excitations, while the VIF fields can be asymmetric only in the offset direction of the excitation position and remain symmetric in other directions. The excitation position is inclined to inhibit the deformation of the thin plate in adjacent regions, thus exhibiting similar effects to wave nodes. With the offset of excitation positions, the deformation of the thin plate, as well as the morphology of the flow fields, becomes complex; the connection and cancellation of the flow fields can be observed, as shown in Fig. 3H. As the excitation position is not symmetric in any direction, the resulting flow fields show no symmetric characteristics with complicated morphologies.

As for excitation frequency, its influence should be considered from 2 aspects. On the one hand, the frequency can change the vibration shapes of the thin plates, thus determining the morphologies of the flow fields. On the other hand, the symmetry is determined by the structure and excitation, and cannot change with the frequencies. As shown in Fig. 3B, the inward flows on the long edges at low frequency are transformed into outward flows at high frequency, while this transformation is symmetric as both sides change at the same time. It means that the frequency itself cannot alter the symmetry of VIF fields. This also applies to the asymmetric situations, as shown in Fig. 3C. The flow fields are always asymmetric in the direction of the dashed red line as the frequency changes from 40 to 55 Hz, but the direction of the flow on the base edge has changed inwardly. With the increase of excitation frequency, the vibration shapes at higher mode will appear, and the produced flow field will be more complex. The complexity of the flow field can affect the locomotion ability due to the vortex; therefore, the vibration shapes at low frequencies with simple deformations exhibit more concise flow fields, resulting in effective locomotion.

According to the analysis above, the generation of asymmetric flow fields is mainly decided by the plate structures and excitation positions, and thus, we can investigate the locomotion strategies for water surface robots focusing on the configurations of these 2 key factors.

### Effective locomotion strategies for robots

The effective vibration excitation is a critical foundation of using the VIF mechanism for locomotion, while the Modal Exciter in the above experiment is not facilitated for integration due to its large size and weight. Thus, a small vibration exciter, ERM (eccentric rotating mass) motor, is selected to apply the VIF mechanism as a locomotion strategy for miniature water surface robots. A detailed analysis about the motor is illustrated in Note S3 and Fig. S3. This exciter can combine with different thin plates easily for its small size, and to determine an effective locomotion strategy based on the VIF mechanism, we adopt several typical configurations with different thin plates and exciter quantities for experiments (see Note S4 and Fig. S4).

**Fig. 4.**
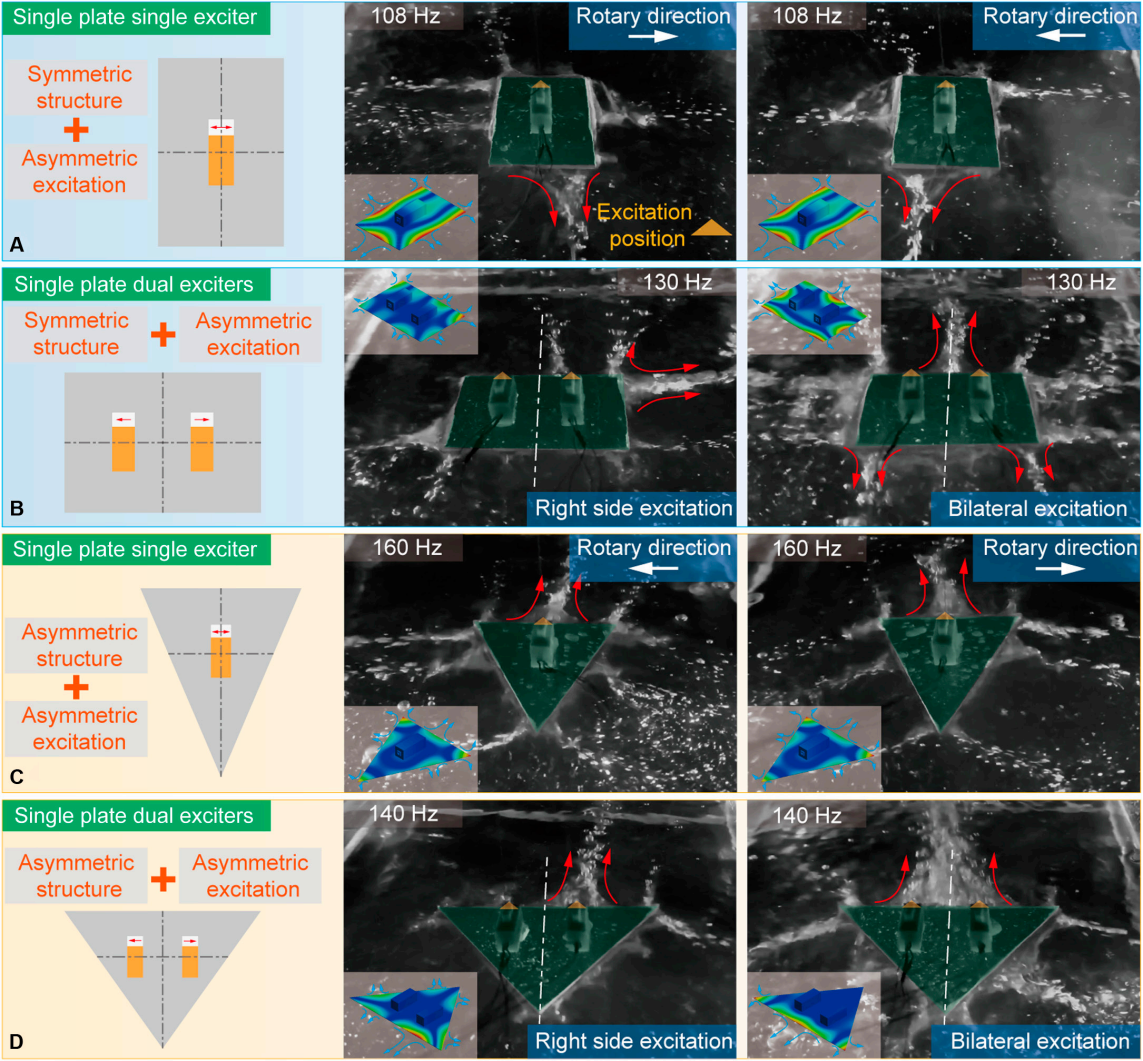
Flow fields generated at different thin plate and exciter configurations. (A) Flow fields generated by a rectangular plate with a single exciter. (B) Flow fields generated by a rectangular plate with dual exciters. (C) Flow fields generated by a triangular plate with a single exciter. (D) Flow fields generated by a triangular plate with dual exciters.

For a rectangular plate with a single exciter, as shown in Fig. 4A, the ERM motor is located at the center, while the actual excitation position is offset (marked by an orange triangle). Then, a concentrated main outward flow (marked by red arrows) is produced at the anti-node of the short edge distal from the excitation position. Although the flow fields still obey the rules of the VIF mechanism, there are some unique features for the flow fields produced by ERM motors. The outward flows are more distinct than inward flows, and the flow fields are greatly influenced by the motor rotary direction, which leads to an obvious deviation of the outward flows. It means that a single ERM motor can control the direction of flow fields through changing its rotary direction. Thus, the locomotion strategy can be adopted by a water surface robot with a single motor: Its rotational motions can be realized by the 2 deviated outward flows, while its linear motions can be achieved via quick switching of the 2 outward flows.

For a rectangular plate with dual exciters, the flow field shows superposition property, as shown in Fig. 4B. The symmetrically arranged motors can generate a deviation outward flow under the unilateral excitation of a single motor. In the case of simultaneous excitation of dual motors, the flow field excited by single motor overlays together and the resultant outward flows are almost balanced (along the center line between 2 motors, marked by white line in Fig. 4B). Then, for the locomotion strategy of a water surface robot with dual motors, its linear motions can be realized by the resultant outward flows excited by 2 motors, while its rotational motions can be achieved by the 2 deviated outward flows under the unilateral excitation. Besides, the outward flows generated by the motors in other offset positions are also analyzed (see Note S5 and Fig. S5), and the results show that they can also be used to realize specific motions by the abovementioned locomotion strategies.

Furthermore, the structure symmetry of the thin plate affects the locomotion effect significantly. The symmetric plates can always generate outward flows in reverse direction, which can produce a compensation in the resultant force. Compared with rectangle plates, the triangular plate can produce divergent outward flows at the angle regions (see Fig. 4C and D), and when the dual motors work together, the triangular plate can produce a distinct outward flow at the base edge, and it is much more furious than that of the rectangular case. These experimental records are shown in Movie S4.

### Development and experiments of robots

To verify the feasibility of the locomotion strategies, 2 miniature water surface robots are developed. One is configured with a triangular plate and dual exciters (named as Robot-I), while the other is designed as a rectangle plate and single exciter (named as Robot-II).

#### Configurations of the water surface robots

**Fig. 5.**
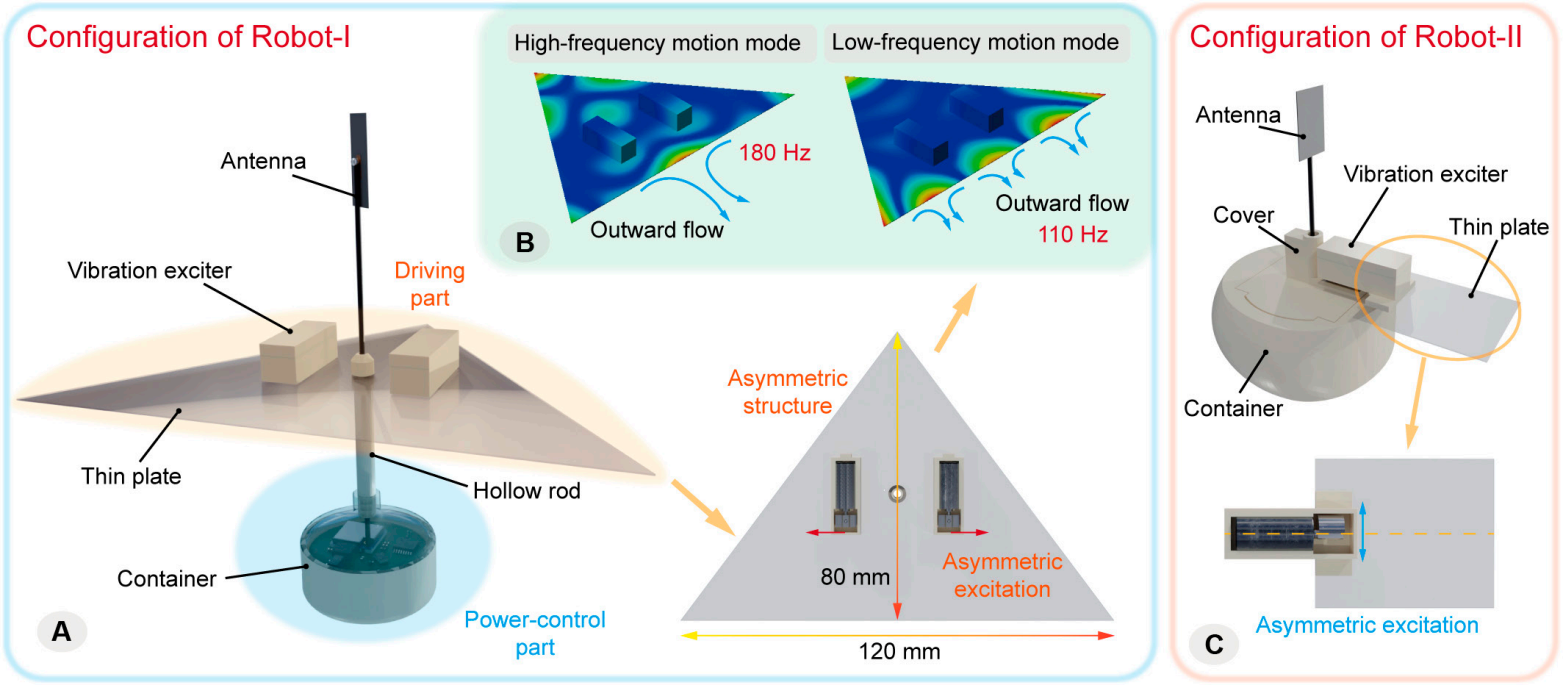
Structure configurations of the water surface robots. (A) Configuration of Robot-I with dual exciters. (B) Effective locomotion modes at 2 vibration frequencies. (C) Configuration of Robot-II with single exciter.

Robot-I contains a driving part and a power-control part, as shown in Fig. 5A, in which the triangular thin plate and 2 ERM motors compose the driving part. The driving part is asymmetric in both structure and excitation. The excitation methods of the dual exciters arranged on both sides will control the rotational and linear motion, while the structural asymmetry will enhance the propulsion effect. The power-control part includes a container integrating the power supply and the control system. A hollow rod is designed to support the antenna for wireless communication. To make the robot stably float on the water surface, the power-control part is designed far below the thin plate to be located underwater, thus lowering the gravity center. The buoyancy force is also adjusted to ensure that the thin plate can be located at the water surface exactly. As for the ERM motor, its maximum frequency is measured to be about 250 Hz, and the harmonic response analysis of the driving part shows that it can produce effective locomotion at 2 vibration shapes in this frequency range. A single concentrated flow perpendicular to the base edge will appear at a higher frequency (typical vibration shape at 180 Hz), and a flow array distributed on the base edge will appear at a lower frequency (typical vibration shape at 110 Hz), as shown in Fig. 5B. Besides, Robot-II also achieves the integration of power supply and control system. It has a simpler structure with only one motor and can move in narrow areas flexibly, as shown in Fig. 5C. Its driving plate is symmetric, while the excitation is asymmetric. Thus, the rotational motion is realized by the deviated outward flows caused by rotary direction, and the linear motion can be realized by the switching of the deviated outward flows.

#### Motion experiments of the water surface robot

The prototype size of Robot-I is about 120 mm × 80 mm × 52 mm, with a mass of 21 g. The voltage exerted on the motor can be adjusted by the pulse width modulation (PWM) method to regulate the excitation frequency. The detail of its integrated control system is shown in Note S6 and Fig. S6.

**Fig. 6.**
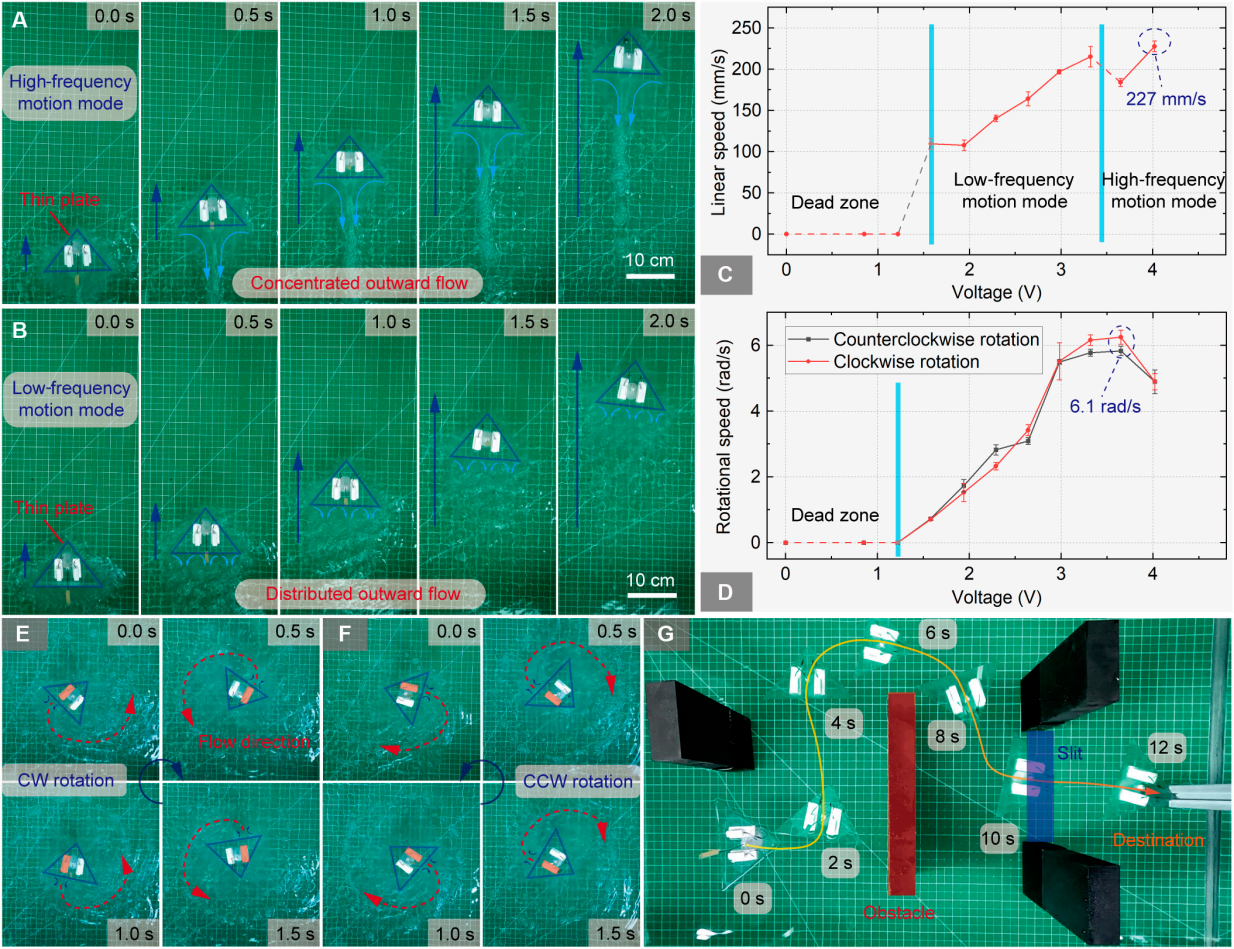
Parameter evaluation experiments of Robot-I. (A) Linear motion experiments with a concentrated outward flow in a water tank. (B) Linear motion experiments with distributed outward flows in a water tank. (C) Linear velocity–voltage curves. (D) Rotational velocity–voltage curves. (E) Clockwise rotational motion experiments in a water tank. (F) Counterclockwise rotational motion experiments in a water tank. (G) Obstacle bypass experiment in a water tank.

The general kinematic characteristics of Robot-I are evaluated in an experimental tank. It can move linearly when dual motors vibrate simultaneously, and different flow fields appear at different frequencies. The linear motion trajectories are shown in Fig. 6A and B, and it can be seen that a concentrated outward flow appears in the middle of the base edge at about 200 Hz, while distributed triple outward flows appear at about 120 Hz, which is consistent with the simulation analysis. The linear velocity–voltage curve is shown in Fig. 6C. The maximum linear velocity reaches about 227 mm/s, and the velocity curve also shows 2 corresponding locomotion segments. The speed of the robot decreases steadily with the decreasing of voltage in each stable motion mode. However, it exhibits an abnormal increase in speed as it switches between the 2 motion modes. Compared with the high-frequency motion mode, the low-frequency motion mode shows triple distributed anti-nodes on the base edges according to simulation, while the deformations around the angle are weaker. Therefore, the propulsion effect produced by this motion mode is better, thus causing the increase of speed as the motion modes are switched, which may also be relevant to the resonant state at this frequency. When a unilateral motor vibrates, an outward flow appears on the base edge just behind the motor, and the robot shows clockwise (CW) and counterclockwise (CCW) rotational motion under the deviated outward flows (see Fig. 6E and F). The tracks of the outward flows are marked by dashed red arrows, and the rotational directions are marked by blue arrows. The rotational velocity–voltage curve is shown in Fig. 6D, in which the left and right rotational speeds are well consistent, and the maximum rotational speed is about 6.1 rad/s. Besides, we construct an environment with obstacles on the water surface in the laboratory to evaluate its pass ability based on the integrated control system. As shown in Fig. 6G, the robot can bypass the obstacles on the water surface and go through the slit area effectively to reach the target destination, thus validating its potential of working in complex environments. The experimental records are shown in Movie S5.

**Fig. 7.**
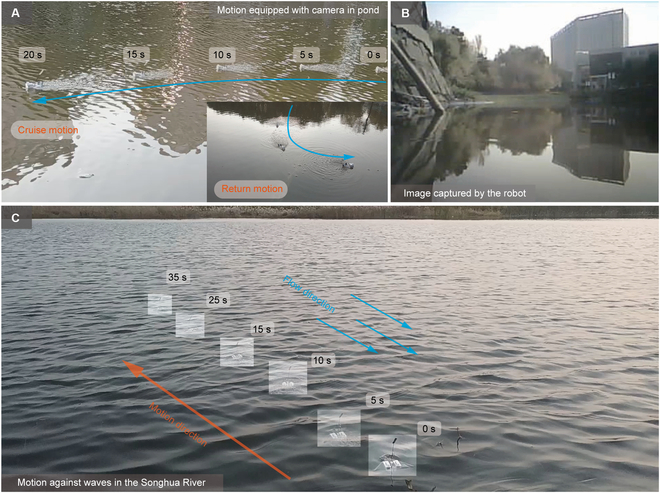
Outfield experiments of Robot-I. (A) Motion experiment in a pond equipped with a camera module. (B) Image captured by the robot in the pond. (C) Motion against waves in the Songhua River.

We further conduct outfield experiments for Robot-I to evaluate its motion ability in real-world environments. An image-capturing test is carried out in a pond, and the motion trajectory is shown in Fig. 7A. A specifically designed miniature camera module is mounted on top of the robot during the motions, whose weight is 13.2 g (about 50% of the robot own weight), and the image frame is shown in Fig. 7B. The experiment shows that Robot-I exhibits stable and controllable movement ability in the outdoor pond, which verifies its application potential, such as information collection (see Movie S6). Furthermore, we place the robot inside the Songhua River (Harbin, China) and conduct a motion experiment on an undulating water surface. The keyframes of the motion trajectory are shown in Fig. 7C. Robot-I exhibits superior motion stability to travel against intense flows and waves (see Movie S7). Besides, we also conduct a series of experiments on Robot-II, and it realizes linear motion with a maximum speed of 109 mm/s. Typically, the robot can go through a narrow pipeline, benefiting from its small size. The corresponding results are provided in Note S7, Fig. S7, and Movie S8.

## Discussion

In this work, we reveal the VIF mechanism of thin plates on the water surface innovatively and investigate the influence of vibration shapes on the flow fields. Then, we analyze the main factors (plate structure and excitation condition) affecting the VIF fields and realize the generation and control of asymmetric flow fields. Furthermore, we establish the locomotion strategies by exploring the flow field variations using ERM motors as exciters. Finally, 2 water surface robots with different plates and exciters based on the VIF mechanism are developed verifying the feasibility of the proposed VIF mechanism and locomotion strategies.

The VIF mechanism is intrinsically an interaction between structural vibration and fluid motion, and the vibrating thin plate itself can be regarded as a transportation source of the fluid clusters through the attraction and exclusion process of nodes and anti-node deformations. Thus, this VIF mechanism not only is capable of robot locomotion but also can be used for other applications, such as the directional movement of small objects or the collection of floating particles by specific flow fields on the water surface.

Compared with the previous underwater or water surface oscillation situations, there are some distinct differences, as reflected in the following aspects. The first difference lies in the deformation of thin plates. The previous flapping or undulation motion strategies are mostly inspired by the caudal fin or flagellum of organisms. These robots usually adopt configurations with narrow and flexible rectangular plates, which can be stimulated to generate bending deformations in traveling or quasi-traveling waveforms. However, we focus on the more general deformation of different thin plates, such as the plates with triangular or trapezoidal shapes, and analyze their influence on the flow fields under specific vibration shapes of the plates as standing waves. The second difference lies in the excitation methods. The previous strategies usually adopt some customized materials or transmission mechanisms to produce oscillation. Some typical methods utilize plates with special magnetization profiles or photothermal characteristics; thus, they can be actuated by external magnetic fields or light power to generate traveling waves. The oscillation frequencies of these strategies are generally below 10 Hz. We use the ERM motor as the excitation source, which has higher excitation intensity and is easier to control. Its excitation frequency is usually above 100 Hz, which is much higher than the current locomotion strategies. This provides totally different insights for high-frequency propulsion in the water surface environment. The third difference lies in the propulsion principle. The propulsion of the flapping or undulation plate is about the generation of vortex in the surrounding fluid. In contrast, the generation of flow fields in the VIF mechanism is due to the surface wave. This also leads to more complex variations of the flow field on the water surface caused by the deformations of the thin plates.

To apply this VIF mechanism to the water surface robots, we first identify the basic properties of the VIF fields that can produce propulsions: the asymmetry of the flow fields. Then, we establish general methods to generate the asymmetric VIF fields by analyzing the 2 main factors, which provide the theoretical foundation for robots. From the other perspective, an integrated vibration exciter is also crucial for water surface robots. Thus, a typical small vibration exciter, the ERM motor, is adopted, and the corresponding locomotion strategies are proposed for the motion control, which provides the realization foundation for the robots.

Regarding the water surface robots developed in this work, the vibration-based locomotion strategies allow a direct drive on the actuators without transmissions such as joints or linkages, which guarantee the small sizes of the robots. Here, we give a comparison between Robot-I and other typical studies [31–40]. From the motion characteristics aspect, the size of Robot-I is comparable to the water strider robots and the Marangoni-driven robots, whereas its linear motion speed on the water surface reaches about 227 mm/s, which is superior to most of the other aquatic robots, as shown in Fig. S8, and a detailed table is shown in Table S1. The robot can also realize fast in situ rotation with a speed of 6.1 rad/s. Meanwhile, it possesses the ability to regulate its speed in a wide range by adjusting the excitation voltage, thus meeting different speed requirements. From the application aspect, the robot exhibits superior locomotion performance in outdoor environments. It is quite a challenge for most water surface robots, such as those driven by magnetic fields or chemicals, to move freely in external environments due to the motion range limitation. This robot is fully integrated owing to the VIF locomotion strategy, and it has an endurance of about 20 min at the highest motion speed, which depends on the mounted power supply and can be further enhanced by replacement with high-capacity batteries.

The developed water surface robot has proved the effectiveness of the VIF mechanism for aquatic locomotion, while there are still some limitations in the research. During the experiment, the equipment for the flow field observation is not professional enough. Although it is adequate for current research, it is not yet possible to grasp the precise characteristics of the flow field morphologies at high speed. Besides, we focus more on the flow fields in still water without considering the flow speed. This facilitates the rule analysis of the flow fields, but the influence of the income speed on the excited flow fields is not that clear. This will be an important topic for further investigation, and it can be instructive for the robot design to increase the motion performances in real environments. As for the VIF locomotion strategies, there are still reverse and lateral flows for current configurations, which decrease the locomotion efficiency. Therefore, the methods to inhibit the unexpected flows as well as enhance the effective outward flows will be of great importance to further increase the motion performance on water surface. From the aspect of applications, the current robot prototypes are not adequate for real tasks, which can be further improved from 2 aspects. On the one hand, the motion ability of the robot prototype should be enhanced, and its own movement state should be monitored, including the self-position monitoring, the distance detection, and the excitation frequency inspection to ensure a stable movement. On the other hand, the relevant external sensing or functional modules should be fabricated, and these modules will be designed based on specific tasks, such as the water quality inspection and image/sound acquisition module. Thus, the robots can be adaptive in wider sceneries. Besides, the capacity of individual robot is limited specifically for the small robot. In future research, a variety of water surface robots can be designed to realize a cluster motion control and then achieve the cruise in a large motion range and the exploration into small areas.

## Materials and Methods

### Experimental methods for observation of VIF fields

A particle image velocimetry (PIV) method is used to observe the flow field variations on water surface, and the experiment is carried out in a light-insulated dark chamber illuminated by a green laser source, as shown in Fig. S9. The polyethylene (PE) powder with a diameter of about 50 μm is used as tracer particles. It is insoluble in water, and its density is lower than water.

In the experiments to investigate the asymmetry of the flow fields, a Modal Exciter (SA-JZ002) is utilized to produce vibration excitation at designated positions and obtain the variation of the flow fields at different frequencies. The frequencies are selected at stable flow fields when the typical inward or outward flows appear during experiments. In the experiments of analyzing the influence of ERM motors on the flow fields, the motors are powered by a direct current (DC)-regulated power supply and sealed by ultraviolet (UV)-curable resin material with a built-in sensor for rotary speed. The plate material used in the experiments is transparent polyvinyl chloride (PVC) with Young’s modulus of 2.77 × 10^9^ Pa.

### Measurement of the rotary speed of ERM motors

The ERM motors need to be sealed when working in the aquatic environment, and then a rotary speed sensing system is designed for ERM motors to ensure the accuracy of the experiment data for flow field investigation. A neodymium magnet is mounted on the eccentric mass of the ERM motor; thus, the rotary signals of the motor can be collected through a hall element. The subsequent process is carried out through the control system so that the rotary speed can be monitored in real time.

### Materials and fabrication of robot prototypes

Robot-I and Robot-II adopt similar materials and fabrication methods. Considering the reduction of cost for fabrication, the support structures of the robots, as well as the shells of the ERM motors, are mainly processed by stereo lithography appearance (SAL) 3-dimensional (3D) printing. The shore hardness is about 76 to 86; the density is about 1.12 to 1.18 g/cm^3^; the elastic modulus is about 2,559 to 2,678 MPa; and the Poisson ratio is about 0.4. These parts are connected by silica waterproof adhesive. The thin plate of Robot-I is selected as a triangular shape with a hollow rod through it and dimensions of 120 mm × 80 mm × 0.32 mm, and the thin plate of Robot-II is selected as a rectangular shape with sizes of 40 mm × 30 mm × 0.32 mm. The plate material is selected as transparent PVC; the density is about 1.25 g/cm^3^; and the elastic modulus is about 2,770 MPa.

### Fabrication of the image capture module

An image-capturing module is specially designed in the outfield experiment for the water surface robot, which is about 27.5 mm × 23.5 mm × 19.7 mm in size and about 13.2 g in mass. The module adopts ESP32 with an OV2640 sensor as the core to realize the image acquisition and the transmission to remote-control software in real time; it can also store the images in the internal micro secure digital (SD) card at the same time.

### Measurement method of motion performances

A motion measurement platform is built in the laboratory to measure the general motion performances of the robot, and a digital camera is used to capture the motion process to evaluate the linear and rotational motion speeds. During the post-processing, a series of motion videos are processed using the frame superposition method to obtain the robot trajectory, and further, the motion speed can be calculated through the length scale (see Fig. S10).

## Data Availability

All data supporting the findings of this study are available in the paper and the Supplementary Materials.

## Supplementary Materials

Supplementary 1 Notes S1 to S7 Figs. S1 to S10 Table S1 Movies S1 to S8
