# Supplementary material for: Vibration-Induced-Flow Mechanism and Its Application in Water Surface Robot
Source: Research (Wash D C). 2024 Aug 9;7:0449. doi: 10.34133/research.0449 (PMC11312888; doi:10.34133/research.0449)
Supplement: Supplementary 1 — Notes S1 to S7 Figs. S1 to S10 Table S1 Movies S1 to S8 [file research.0449.f1.zip › Revised Supplementary Materials-non-highlighted.docx]

Supplementary Materials

**Vibration-induced-flow mechanism and its application in water surface robot**

Dehong Wang†, Shijing Zhang†, Jing Li†, Haoxuan He, Junkao Liu, Weishan Chen, Jie Zhao, Jie Deng*, Yingxiang Liu*

Email: dengjie21@hit.edu.cn (J. D.), liuyingxiang868@hit.edu.cn (Y.L.).

**The PDF file includes:**

Supplementary Note S1 to S7

Fig. S1 to S10

Table S1

**Other Supplementary Materials includes the following:**

Movie S1 to S8

Supplementary Note

**Note S1. Plate theory of the VIF mechanism**

As far as VIF situation, the plates are homogeneous and isotropic, and the thicknesses are far less than the in-plane dimension, so they can be treated as thin plates, and we can assume that the points on the middle surface experience no parallel displacements according to plate theory of elastic mechanics, as shown in Fig. S1. If analyzed from wave propagation perspective, the transverse deformation of the thin plate excited by the vibration is essentially a plate bending wave, and the wave propagation satisfies the Equation 1:

$D\left(\frac{\partial^{4}w}{\partial^{4}x}+2\frac{\partial^{4}w}{\partial^{2}x\partial^{2}y}+\frac{\partial^{4}w}{\partial^{4}y}\right)+m\frac{\partial^{2}w}{\partial^{2}t}=q$ (1)

where *D* is the bending stiffness of the plate, *w* is the transverse displacement of the plate, *m* is the mass per unit area of the plate, and *q* is the transverse load.

For a thin plate with finite size, the different vibration modes can be regarded as different standing waves with definite frequencies, and the overall vibration pattern of the plate is the superposition of different mode shapes, so the solution can be taken as the equation by separating the variables, as show in Equation 2:

$w=\sum_{m=1}^{\infty} T_{m}\left(t\right)W_{m}\left(x,y\right)$ (2)

where *T_m_*(*t*) is the part that changes with time, and *W_m_*(*x*,*y*) represents the mode shape function of the plate; and both parts of the solution can be expressed as a combined form of trigonometric functions.

However, the dynamic modal above is suitable for small deflection situations, and as for the VIF situation when the ERM motor is utilized as vibration exciters, the transverse deflection is of the same order as the plate thickness. Hence it should be treated as a nonlinear problem according to the plate theory, in other words von Kármán plate. The governing equation of the thin plate excited by vibration can be derived as Equation 3:

$D\nabla^{4}w+m\frac{\partial^{2}w}{\partial^{2}t}=\delta\left(\frac{\partial^{2}\Phi}{\partial x^{2}}\frac{\partial^{2}w}{\partial y^{2}}-2\frac{\partial^{2}\Phi}{\partial x\partial y}\frac{\partial^{2}w}{\partial x\partial y}+\frac{\partial^{2}\Phi}{\partial y^{2}}\frac{\partial^{2}w}{\partial x^{2}}\right)+q$ (3)

where *D* is the bending stiffness of the thin plate, *w* is the transverse deflection, *m* is the mass per unit area, *q* is the transverse load, and *Φ* is stress function satisfying the Equation 4:

$\nabla^{4}\Phi=E\left[\left(\frac{\partial^{2}w}{\partial x\partial y}\right)^{2}-\frac{\partial^{2}w}{\partial x^{2}}\frac{\partial^{2}w}{\partial y^{2}}\right]$ (4)

where *E* is Young’s modules of the thin plate. It is difficult to obtain an accurate analytical solution for this equation; therefore, we will use simulation analysis to determine the specific deformation of the thin plates, and the vibration modes of the plates can also be regarded as standing waves in natural frequencies.

**Note S2. Flow field of other thin plates with typical shapes**

The flow field morphologies produced by some other typical thin plates are investigated, including the regular triangle, the regular hexagon, and the ellipse. The plates are also excited by a harmonic vibration at the geometric centers, and the flow fields generated by these plates also conform with the basic rules of VIF mechanism.

For the regular triangle and regular hexagon, the excited flow fields are consistent on all edges at low frequency (see Fig. S2A-B); but due to the obtuse inner angles of the hexagon, it is actually more difficult to be deformed as wave anti-node than the triangle. Besides, the flow field excited by a hexagon is much more complex with the number of edges increases. It means that they are generally not conducive to be used for locomotion. In the case of ellipse, the excited flow fields also obey the VIF mechanism for the curved edges, as shown in Fig. S2C. Since the major and minor axes of the ellipse differ greatly, the overall deformation in the two directions can be generated, thus the inner and outer flows along major axis can be produced at different frequencies respectively.

**Note S3. Dynamic analysis of the eccentric rotating mass motor**

In terms of excitation sources, the small vibration exciters mainly include piezoelectric material, ERM motors, LRA motors, and coin vibration motors. Piezoelectric excitation generally requires specific excitation signals with high voltage, which is difficult to integrate, and the vibration strength is relatively weak. The LRA motors are excited by AC signals, which makes it difficult to control. Besides, the motors can only produce strong vibration when resonating at specific excitation frequencies; while the vibration is much weaker at the rest of the frequencies, which cannot ensure the smooth speed adjustment. The coin vibration motor and the ERM motor utilize similar principles with different structural features. The selection is mainly based on the design of the robot prototype. Therefore, the ERM motor with simple control methods and higher excitation strength is selected in this work.

Eccentric rotating mass (ERM) vibration motor is a typical electric vibration exciter. It can be excited by direct current and produce vibration excitation via the rotation of an eccentric mass, as shown in Fig. S3A-B. As for other vibration exciters, such as linear resonant actuator, the excitation method are more complicated and the vibration intensity are weaker, which are not appropriate for a fast water surface locomotion.

The ERM motor will generate a centrifugal force *F*(*t*) when rotating at angular velocity of *ω* (see Fig. S3C), which can be obtained as Equation 5:

$\left\{\begin{matrix} F\left(t\right)=F_{0}e^{i\omega t} \\ F_{0}=md\omega^{2} \end{matrix}\right.$ (5)

where *F*_0_ is the amplitude of centrifugal force, *m* is the mass of the eccentric rotor, *d* is the eccentric distance. It shows that the vibration force is in a quadratic relationship with the angular velocity, as well as the vibration frequency, which determines the vibration amplitude; and the angular velocity is positively related with the input voltage. Therefore, the vibration amplitude is fully coupled with the frequency.

The direction of the centrifugal force is outwards along the direction of the rotational center and the mass center of eccentric rotor, so the horizontal and vertical components *F*_x_ and *F*_y_ of the centrifugal force can be derived as Equation 6:

$\left\{\begin{matrix} F_{x}\left(t\right)=F_{0}\cos\left(\omega t\right) \\ F_{y}\left(t\right)=F_{0}\sin\left(\omega t\right) \end{matrix}\right.$ (6)

Due to the features of the ERM motor, the excitation shows the feature of elliptical vibration. Therefore, the influence of the lateral harmonic excitation *F*_x_, in other words, the rotational direction of the motor, also needs to be considered except for the excitation position, when analyzing the asymmetric flow field with an ERM motor as the exciter.

**Note S4. Basic configurations for effective locomotion**

When the ERM motors are used as exciters, a variety of configurations can be obtained by the combinations of exciters and thin plates, as shown in Fig. S4. The combinations vary with the shapes of thin plates and the number and position arrangement of exciters.

In the main manuscript, the typical configurations of thin plates with exciters located at center are selected to give a detailed illustration, as shown in Fig. S4A-B. For configurations of single exciter, the rectangular and triangular shape plates are adopted with the same area, respectively. The ERM motor is arranged longitudinally on the symmetrical axis and coincide with the geometrical center of the plate, in which the excitation position and direction are asymmetric. For configurations of dual exciters, the plates also adopt the rectangular and triangular shapes with the same areas, and the dual exciters are placed parallel on both sides of the symmetrical axis, thus the positions and the quantities of exciters are designed for asymmetrical excitation, and the cooperation of the dual exciters will be realized.

As the positions of exciters are offset, the configurations can be more complex, as shown in Fig. S4C. In the case of single exciter, the exciter can have longitudinal offset arrangement and horizontal offset arrangement. In the case of dual exciters, the exciters can have parallel offset arrangement and parallel reversed arrangement on both sides of the axis. The experiments for these four cases are conducted for comparation with symmetric located cases.

**Note S5. Flow field produced by ERM motors at offset positions**

We explored the produced flow fields when the exciters are arranged in offset positions, as shown in Fig. S4C.

For the configurations of single rectangular plate with single exciter, we evaluate the flow fields under two offset positions (the longitudinal offset arrangement and the horizontal offset arrangement), as shown in Fig. S5A-B, and the produced flow fields still conform to the basic rules of VIF mechanism. Taking the longitudinal axis offset situation as an example, the thin plate produces obvious outward flows at the anti-node regions, and the strength of the flow on one side edge is distinct due to the lateral excitation of the exciter. Compared with the VIF fields when the exciter is located at symmetric position, the flow fields are significantly inhibited in offset direction, while a more obvious flow is produced in the opposite direction. In the horizontal offset arrangement, the reverse flow is almost inhibited, as shown in Fig. S5B. However, this configuration cannot modify the directions of the flow fields effectively through rotary directions of motors.

For the configuration of single rectangular plate with dual exciters, we also evaluate the flow fields under two typical offset positions, which are the parallel offset arrangement and the parallel reversed arrangement on both sides of the axis, and the results show the superposition phenomena in the case of dual exciters. As both exciters are offset on one base side of the plate, the outward flow of this side edge is indeed inhibited, but no obvious outward flow appears on the other side as expected, instead extremely obvious outward flows appear on both side edges, as shown in Fig. S5C. When the dual exciters are reversely arranged, the excitation positions are center symmetric, so that the flow field produced by each exciter is asymmetric in the same direction. The excitation of dual exciters is equivalent to the enhancement of the single one, as shown in Fig. S5D, and it still can only generate rotational motion.

**Note S6. Design of the control system of the water surface robot**

Robot-I and Robot-II have similar control systems, and the control system of the robots can be divided into a remote-control terminal and an on-board controller. The framework of the control system is shown in Fig. S6.

The remote-control terminal is a software platform developed based on C#, as shown in Fig. S6A. The software can realize the basic communication with the on-board controller to send instructions and receive information in real time. A custom communication protocol is designed to format the commands and ensure the standardization of transmission. The graphical user interface of the software is designed by the UWP API, and the communication connection panel is shown in Fig. S6A, in which the real-time information list is displayed below. The voltage exerted on ERM motors are displayed on the control panel in real time, and all the commands are encapsulated inside the program. The external image capturing module adopts sensor of OV2640 and MCU of ESP32-C3 to achieve remote wireless image transmission. It establishes connection with the remote-control terminal through Wi-Fi and uses the UDP protocol to complete image transmission without handshake protocol, ensuring the real-time requirement. The image acquisition interface is shown in Fig. S6A, with a display window on the right and configuration options on the left. The quality and size of the video can be controlled, and the image can be downloaded to the local SD card.

The on-board controller comprises a control part (consists of a micro-control unit and a communication unit) and a driving part (consists of a driving unit, an actuation unit, and a power supply unit). The hardware of the control system is determined according to the function requirements in different motion conditions. The micro-control unit is selected as STM32F103 series and is served as the core of the control system, which is responsible for information receiving and instruction converting. The communication unit adopts the Bluetooth Low Energy (BLE) module, and an antenna is used to lead out the signal, which acts as the interface for the bidirectional information transmission. The driving unit adopts DRV8833 motor driver equipped with two identical H-bridges, and the output voltage can be controlled through PWM signal. The actuation unit is a coreless ERM motor, and the rotational speed-voltage curve is shown in Fig. S6C, in which the maximum excited frequency is about 250 Hz. The control part and driving part of the robot are integrated completely, and the integrated control system is designed, as shown in Fig. S6B. The power supply unit will provide energy for the whole on-board controller. A power management module IP5306 is added to ensure the stability of power supply cooperating with a voltage regulator chip. Thus, the stable movement of the robot can be realized. The capacity of the integrated power supply module decides the endurance of the robot and further determines the maximum traveling displacement. Considering the size requirement of the control part, the battery of 200 mAh is selected for the current prototype, and the measured endurance time is about 20 min at maximum speed, where the maximum distance in the water is about 270 meters. However, the endurance can be elongated by increasing the battery capacity. Besides, the arrangement of photovoltaic materials on the surface of the robot may achieve charging in real-time, thus guaranteeing a long-time operation in further practical applications.

**Note S7. Experiments of Robot-II**

For configuration of rectangular plate with single exciter, we investigate two typical situations: longitudinal offset arrangement and horizontal offset arrangement. To generate deviated outward flow effectively and realize multi-DOF motion on water surface through single exciter, the longitudinal offset arrangement is adopted, as shown in Fig. 4C.

Through harmonic response analysis, the positions of wave anti-node are offset with the rotary directions of motor, as shown in Fig. S7A-B. Thus, the rotational motion of the Robot-II in different directions can be realized, and the trajectories of the counterclockwise and clockwise rotations are shown in Fig. S7C-D. The linear motion is achieved by repeated switching of the motor rotary direction, as shown in Fig. S7E, and the time duty ratio of the motor rotary direction can be adjusted through control system. The velocity-voltage curve is obtained by adjusting the voltage applied to the ERM motor, as shown in Fig. S7F, and the maximum motion speed is about 109 mm/s, which is about half of the linear speed of Robot-I with dual exciters (about 223 mm/s). The experiment records are shown in movie S8.

The main size of Robot-I is only 48 mm × 51 mm × 32 mm. Therefore, it has a superior capability to pass through obstacles. We carry out a pipeline passing experiment for the robot, and its movement trajectory is shown in Fig. S7G. The robot can pass through a pipeline with diameter of 11 cm smoothly, which demonstrates its potential in the applications of inspection for narrow pipes. We also evaluate the motion of the robot in the external environment, as seen in Fig. S7H, but the robot can be affected by the interference on the water surface.


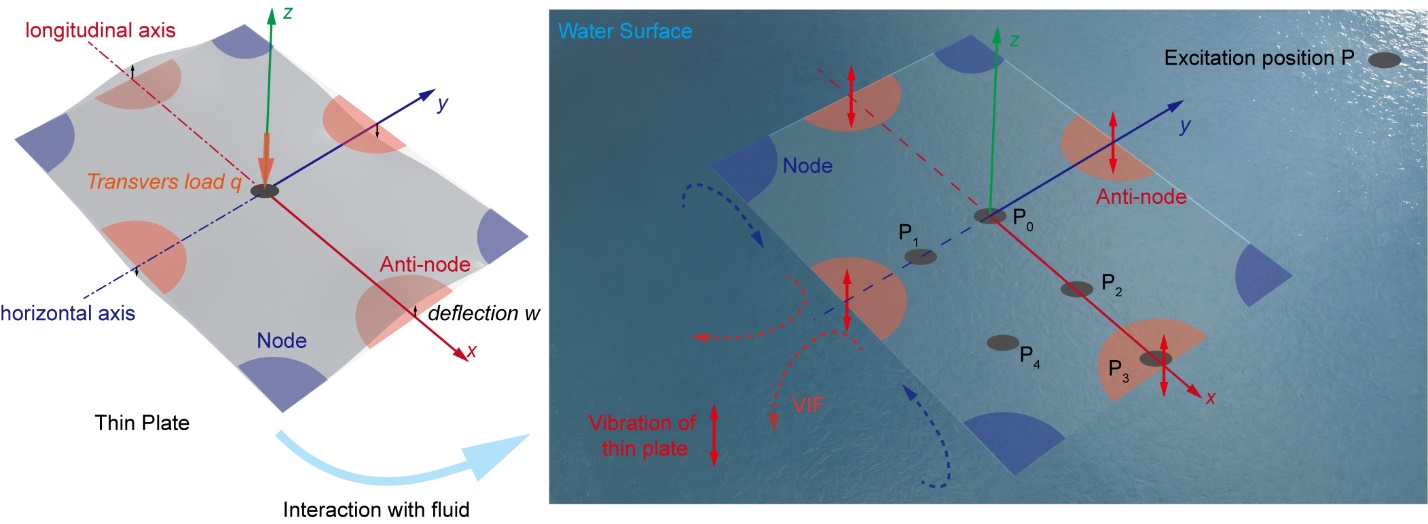


Fig. S1. Vibration excitations and deformations of thin plates.


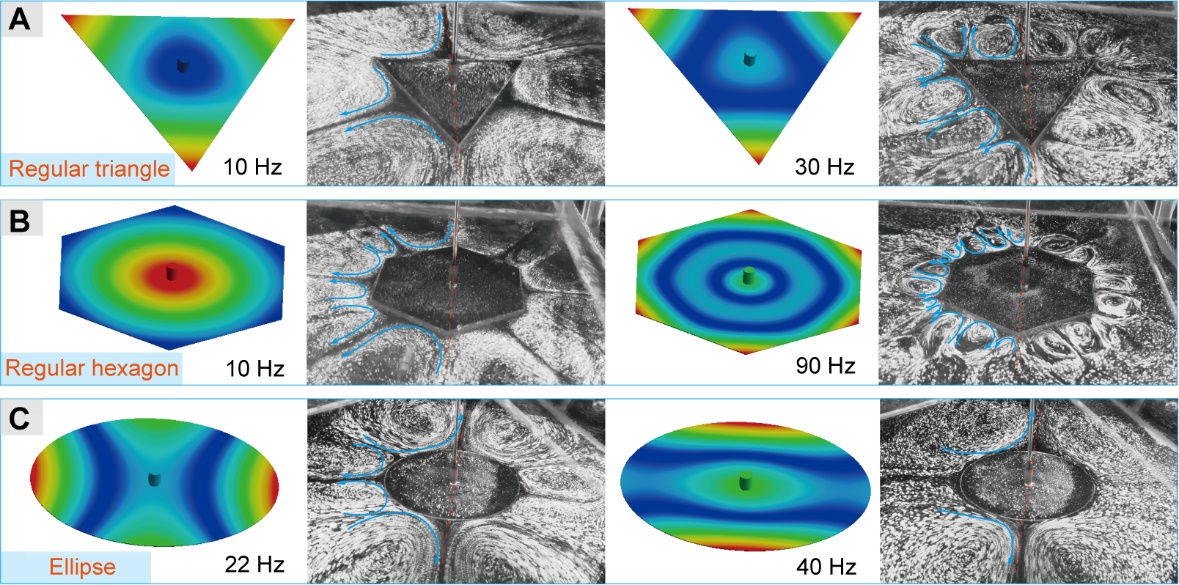


Fig. S2. Flow fields generated by thin plates with the increase of edge number. (A) Flow fields generated by regular triangular plate. (B) Flow fields generated by regular hexagon plate. (C) Flow fields generated by ellipse plate.


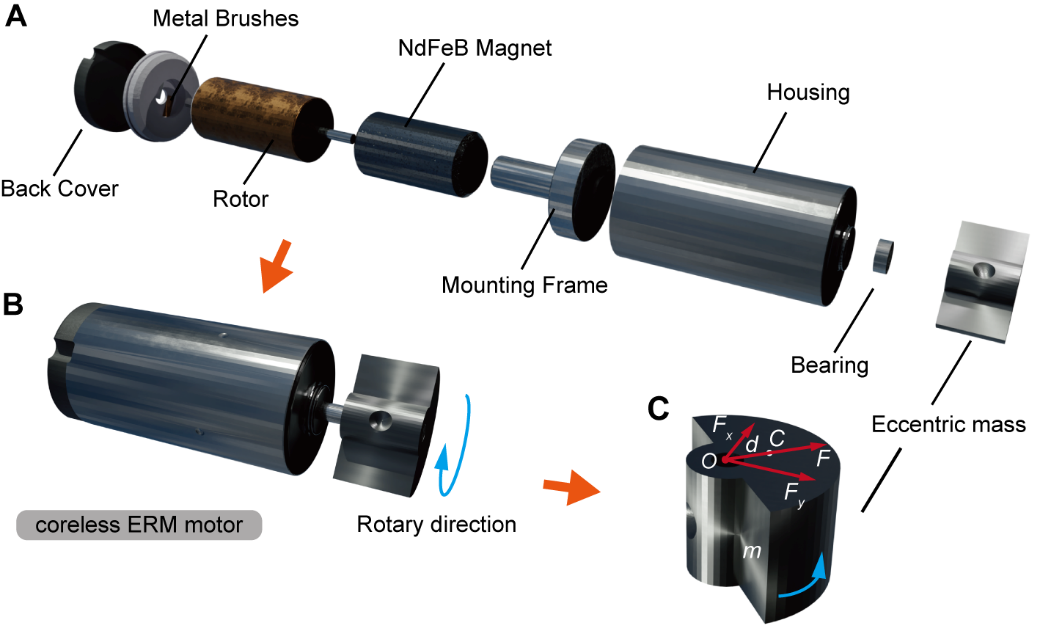


Fig. S3. The basic structure of eccentric rotating mass vibration motor. (A) Inner structure of the ERM motor. (B) Outer structure of the ERM motor. (C) Structure of eccentric rotating mass.


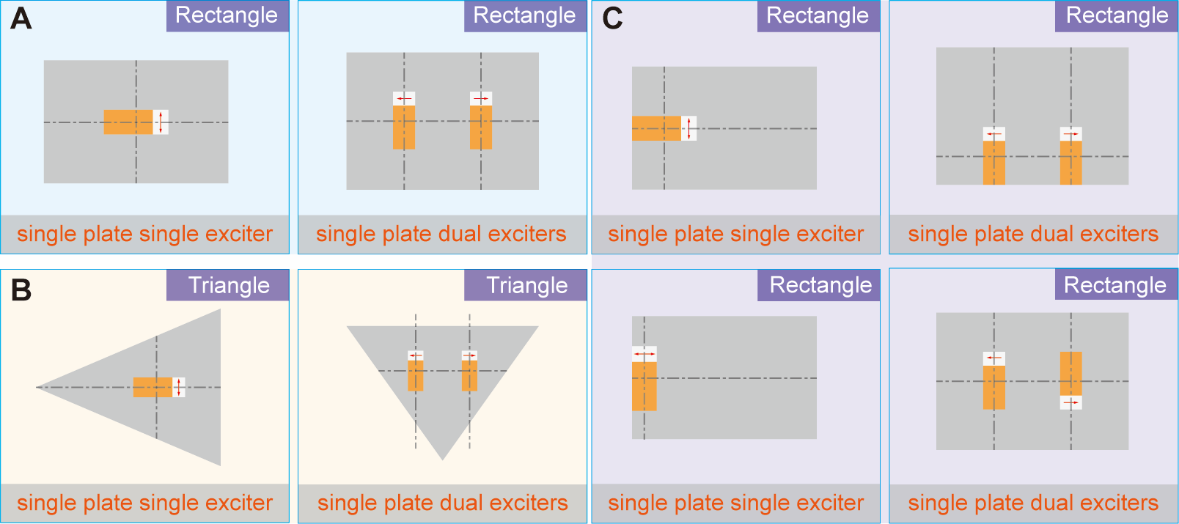


Fig. S4. Configurations of the exciters and the plates for water surface locomotion. (A) Centrally located exciter on rectangular plates. (B) Centrally located exciters on triangular plates. (C) Offset located exciters on rectangular plates.


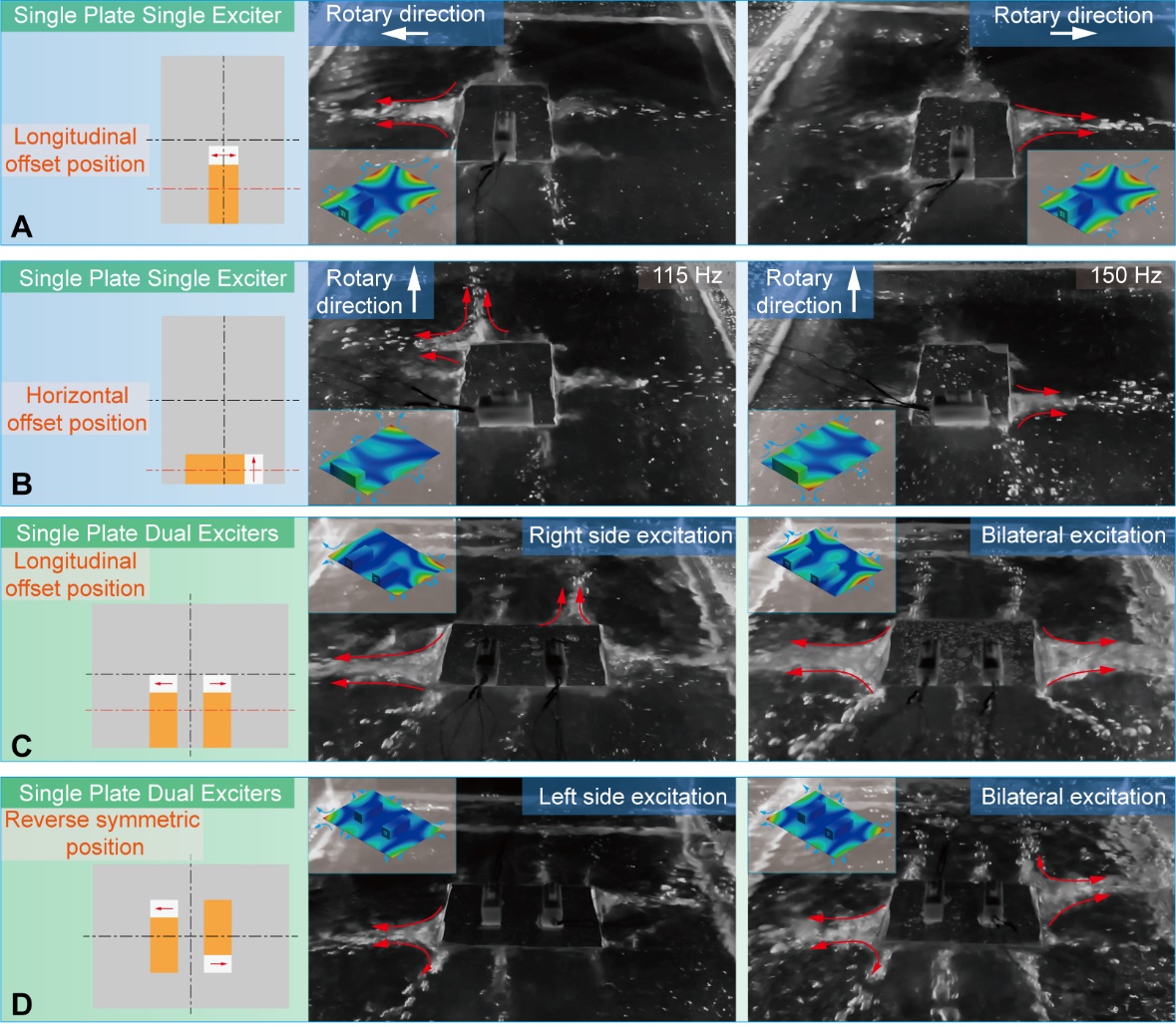


Fig. S5. Flow fields generated by the ERM motors in offset excitation positions. (A) The flow fields when the exciter is longitudinal offset arranged. (B) The flow fields when the exciter is horizontal offset arranged. (C) The flow fields when the dual exciters are parallel offset arranged. (D) The flow fields when the dual exciters are parallel reversed arranged.


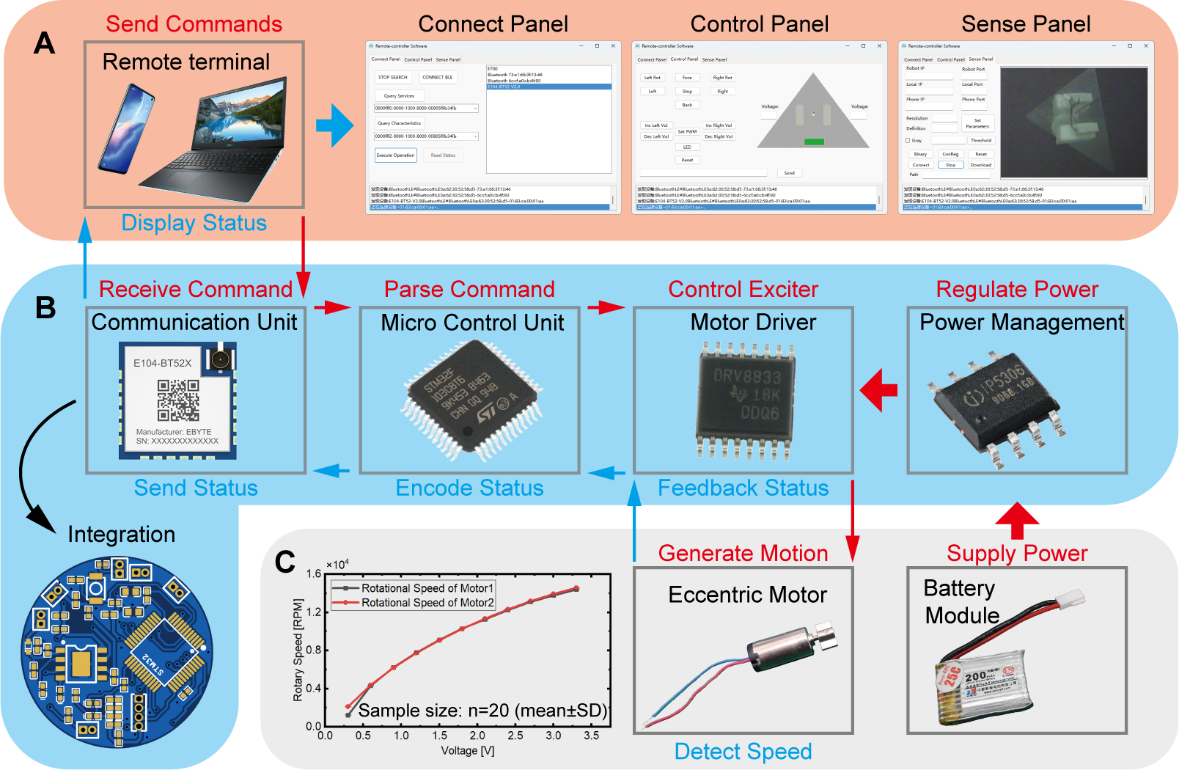


Fig. S6. The framework of the control system of the water surface robots. (A) Remote-control software and the UI design. (B) The on-board controller and the integrated design. (C) The power supply and actuation of the control system.


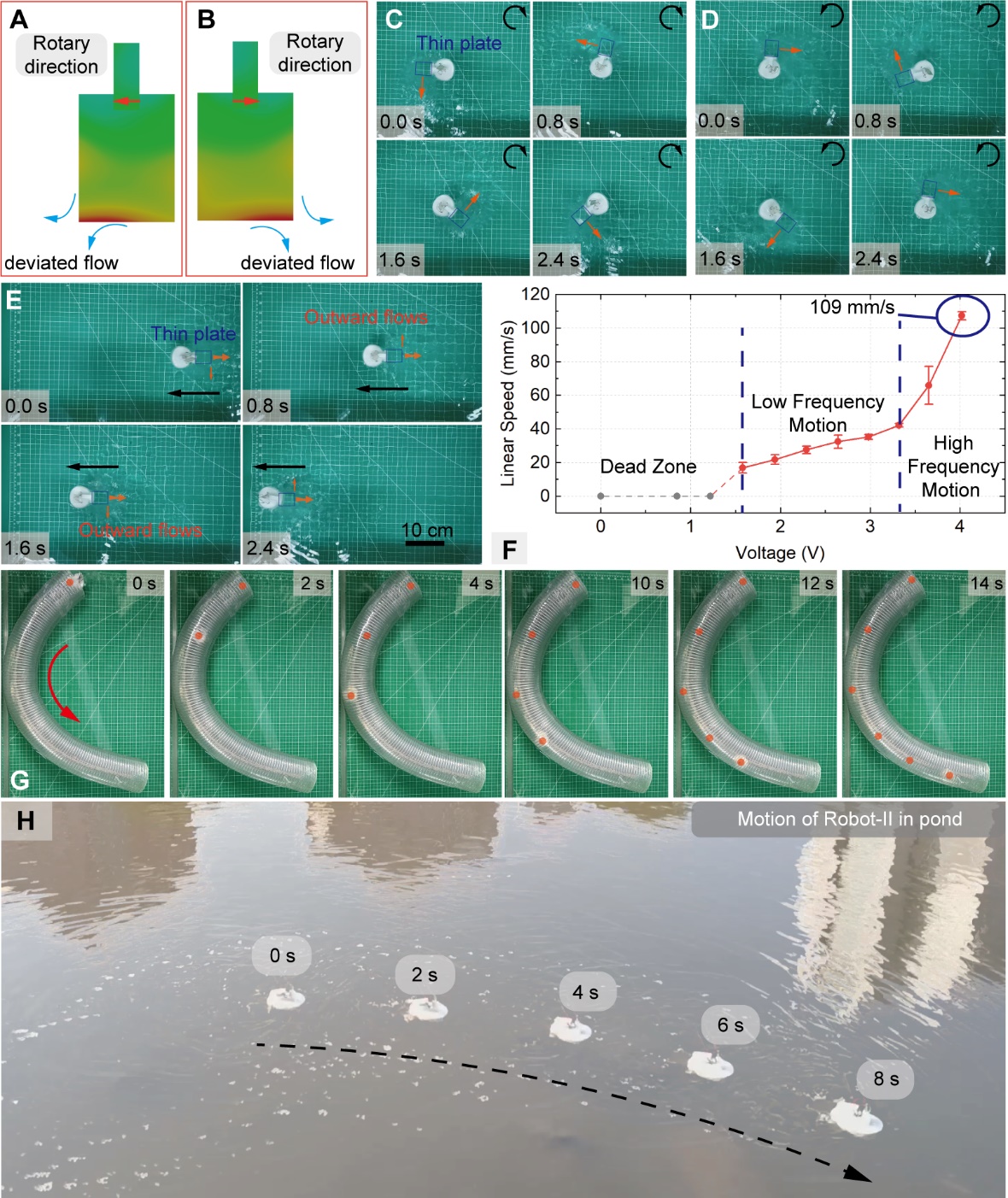


Fig. S7. Motion experiments of Robot-II. (A) Simulation of deviated outward flow in left direction. (B) Simulation of deviated outward flow in right direction. (C) Clockwise rotational motion of Robot-II. (D) Counterclockwise rotational motion of Robot-II. (E) Linear motion of Robot-II. (F) Linear speed-voltage curve of Robot-II. (G) Pipeline through experiment of the robot. (H) Outfield motion experiment of the robot.


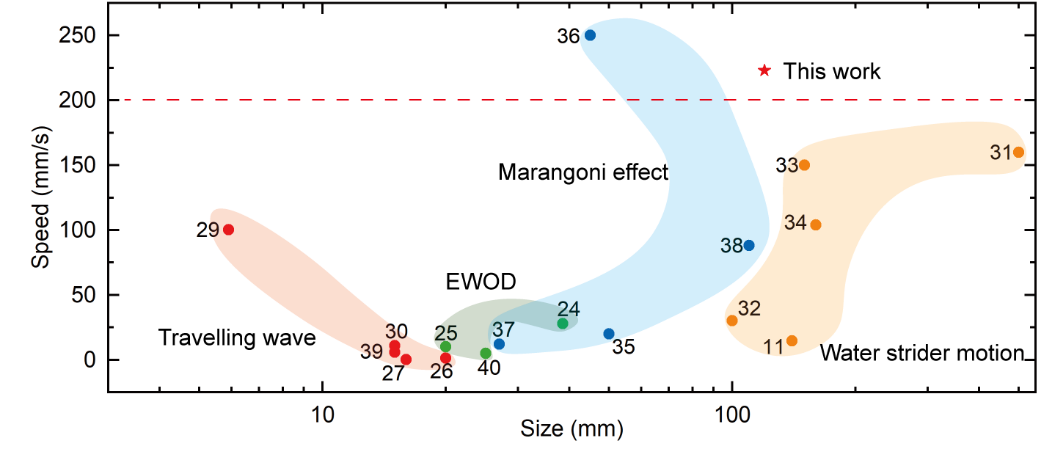


Fig. S8. The speed comparison with other typical water surface robots.


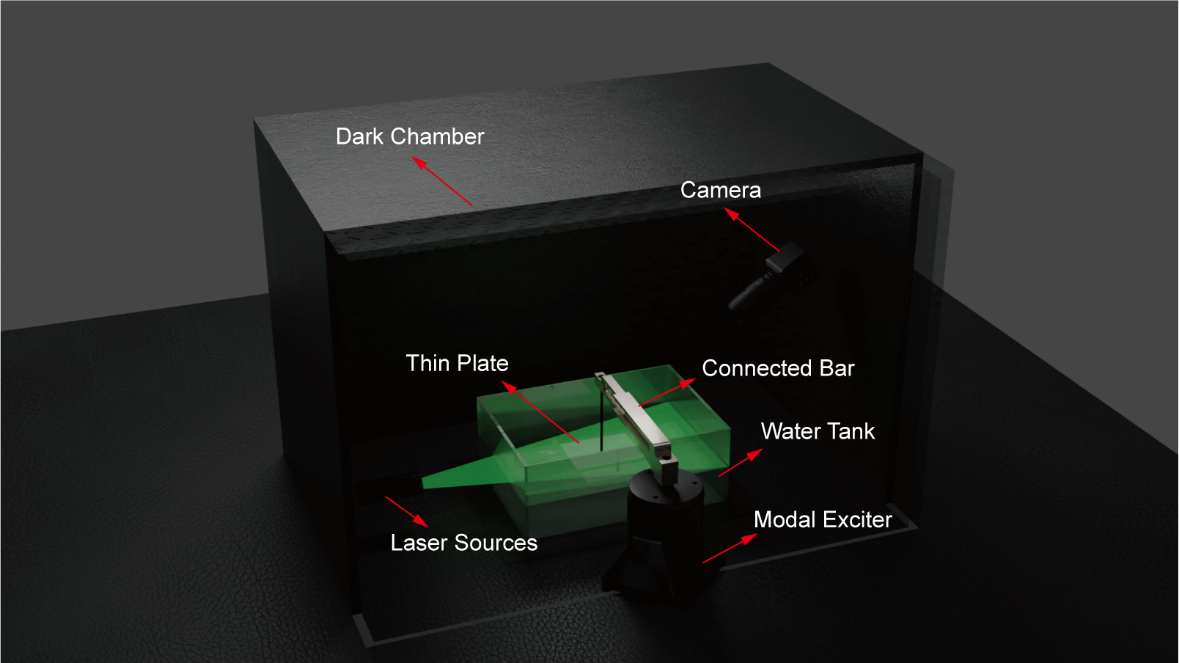


Fig. S9. Experiment scene for observation of the VIF fields.


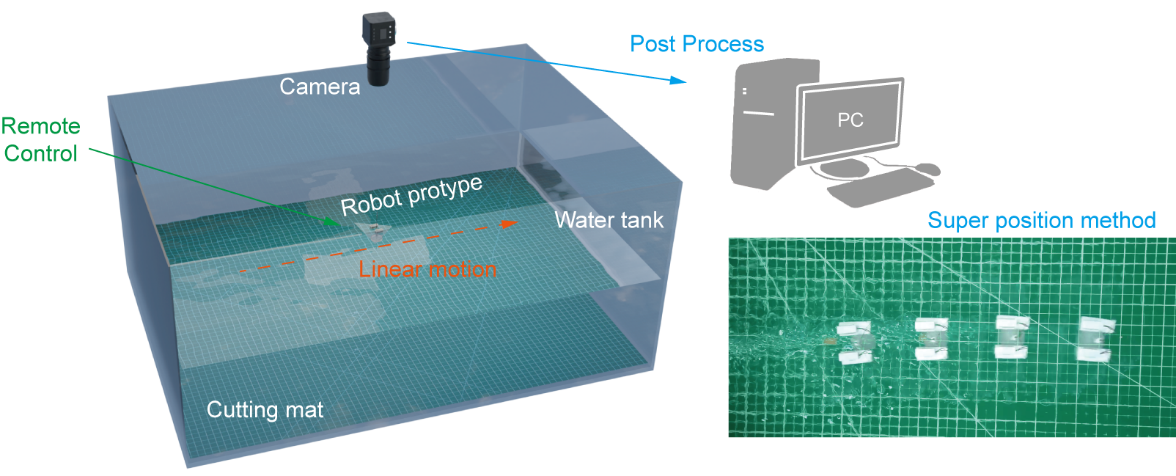


Fig. S10. Experiment platform for motion evaluation of the water surface robots.

Table S1. Motion parameters of the water surface robots in recent research.

| Water surface robot | Years | Locomotion modes | Speed (mm/s) | Size (mm) |
| --- | --- | --- | --- | --- |
| This work | 2023 | Vibration induced flow | 223 | 120×80×0.3 |
| 31 | 2015 | Surface tension | 160 | Supporting leg: 500 |
| 32 | 2007 | Surface tension | 30 | Supporting legs: 50(×4) |
| 33 | 2011 | Surface tension | 150 | 150×50 |
| 11 | 2019 | Surface tension | 14.5 | 140 |
| 34 | 2016 | Surface jump | 104 | 160×140 |
| 35 | 2012 | Marangoni effect | >20 | <50 |
| 36 | 2019 | Marangoni effect | 250 | 45 |
| 37 | 2022 | Marangoni effect | 12.18 | 27×6×5 |
| 38 | 2021 | Marangoni effect | 88 | 110 |
| 26 | 2021 | Light-powered | ~1.3 | ~20 |
| 27 | 2019 | Light-powered | 0.305 | 16 |
| 39 | 2022 | Light-powered | ~5.7 | Actuator: 15×5×0.03 |
| 29 | 2014 | Magnetiztion | 100.3 | 5.9 |
| 30 | 2016 | Magnetiztion | 11 | <15 |
| 24 | 2018 | EWOD | 28 | ~38.6 |
| 25 | 2020 | EWOD | 10 | 10×20×8 |
| 40 | 2009 | EWOD | 5 | 25×10×10 |

Movie S1.

Influence of the localized deformations of thin plates on the VIF fields.

Movie S2.

Flow fields generated by thin plates with typical shapes.

Movie S3.

Flow fields generated by thin plates with asymmetric excitations.

Movie S4.

Flow fields generated by thin plates with ERM motors as vibration exciters.

Movie S5.

Motion experiments of Robot-I in laboratory.

Movie S6.

Outfield motion experiment of Robot-I in a pond with an image capturing device.

Movie S7.

Outfield motion experiment of Robot-I in the Songhua River against the wave.

Movie S8.

Motion experiment of Robot-II (with a rectangle plate and single exciter).
